# Essential role of the histone lysine demethylase KDM4A in the biology of malignant pleural mesothelioma (MPM)

**DOI:** 10.1038/s41416-021-01441-7

**Published:** 2021-06-04

**Authors:** Moshe Lapidot, Abigail E. Case, Ellen L. Weisberg, Chengcheng Meng, Sarah R. Walker, Swati Garg, Wei Ni, Klaus Podar, Yin P. Hung, Ruben D. Carrasco, Aine Knott, Prafulla C. Gokhale, Sunil Sharma, Alex Pozhitkov, Prakash Kulkarni, David A. Frank, Ravi Salgia, James D. Griffin, Srinivas V. Saladi, Raphael Bueno, Martin Sattler

**Affiliations:** 1 Department of Surgery, Brigham and Women’s Hospital, Boston, MA USA; 2 Department of Medical Oncology, Dana-Farber Cancer Institute, Boston, MA USA; 3 Department of Medicine, Harvard Medical School, Boston, MA USA; 4 Department of Molecular, Cellular, & Biomedical Sciences, University of New Hampshire, Durham, NH USA; 5 Department of Internal Medicine, Karl Landsteiner University of Health Sciences, Krems an der Donau, Austria; 6 Department of Pathology, Brigham and Women’s Hospital, Boston, MA USA; 7 Department of Oncologic Pathology, Dana-Farber Cancer Institute, Boston, MA USA; 8 Experimental Therapeutics Core and Belfer Center for Applied Cancer Science, Dana-Farber Cancer Institute, Boston, MA USA; 9 Translational Genomics Research Institute, Phoenix, AZ USA; 10 Department of Medical Oncology and Therapeutics Research, City of Hope, Duarte, CA USA; 11 Department of Information Sciences—Beckman Research Institute, City of Hope, Duarte, CA USA; 12 Department of Otolaryngology Head and Neck Surgery, Massachusetts Eye and Ear Infirmary, Boston, MA USA; 13 Broad Institute of MIT and Harvard, Boston, MA USA

**Keywords:** Mesothelioma, Biochemistry, Cell biology

## Abstract

**Background:**

Malignant pleural mesothelioma (MPM) is a highly aggressive cancer with a dismal prognosis. There is increasing interest in targeting chromatin regulatory pathways in difficult-to-treat cancers. In preliminary studies, we found that KDM4A (lysine-specific histone demethylase 4) was overexpressed in MPM.

**Methods:**

KDM4A protein expression was determined by immunohistochemistry or immunoblotting. Functional inhibition of KDM4A by targeted knockdown and small molecule drugs was correlated to cell growth using cell lines and a xenograft mouse model. Gene expression profiling was performed to identify KDM4A-dependent signature pathways.

**Results:**

Levels of KDM4A were found to be significantly elevated in MPM patients compared to normal mesothelial tissue. Inhibiting the enzyme activity efficiently reduced cell growth in vitro and reduced tumour growth in vivo. KDM4A inhibitor-induced apoptosis was further enhanced by the BH3 mimetic navitoclax. KDM4A expression was associated with pathways involved in cell growth and DNA repair. Interestingly, inhibitors of the DNA damage and replication checkpoint regulators CHK1 (prexasertib) and WEE1 (adavosertib) within the DNA double-strand break repair pathway, cooperated in the inhibition of cell growth.

**Conclusions:**

The results establish a novel and essential role for KDM4A in growth in preclinical models of MPM and identify potential therapeutic approaches to target KDM4A-dependent vulnerabilities.

## Background

Malignant pleural mesothelioma (MPM) is a rare cancer related to the inhalation of asbestos fibers. Asbestos use has not been extinguished globally and exposure persists due to international travel, trade, military-deployments and abatement. MPM can also result from exposure to radiation therapy and is occasionally idiopathic.^1^ The disease is aggressive and highly malignant with median overall survival of only 8–12 months.^2^ MPM tends to cause death by invasive growth of tumour cells and compression of mediastinal structures rather than metastatic growth.^3–5^ Standard therapy is limited to very few therapeutic options, which have at best 40% response rates. Patients diagnosed with MPM mainly receive the cytotoxic drug cisplatin in combination with the anti-folate pemetrexed, which is to date the most effective combination chemotherapy in MPM^6,7^ and may further be combined with bevacizumab.^8^ Recently, immunotherapy, such as nivolumab plus ipilimumab, has also shown efficacy in some MPM patients and has been approved in the USA.^9^ However, the overall prognosis is dismal and there is a significant need for effective long-term treatments.

The genomic landscape in MPM has been defined through whole exome sequencing, revealing predominantly recurrent loss-of-function mutations in tumour suppressors, including *BAP1*, *DDX3X*, *NF2*, *TP53*, *SETD2* and *ULK2*, as well as copy number losses in *BAP1*, *NF2*, *CDKN2B*, *LATS2*, *LATS1* and *TP53*, copy number gains in *RPTOR* and *BRD4* or other genomic alterations.^10^ Additional significant alterations in *LATS2* have been found as well.^11^ SETD2 is of particular interest, as it is required for trimethylation of histone H3 lysine K36, whereas other methyltransferases can lead to mono- or dimethylation of the same residue.^12,13^ In preliminary studies, we found that, while not known to be mutated, KDM4A is overexpressed in MPM. KDM4A regulates H3K36me3 demethylation, but it also reduces levels of H3K36me2 as well as H3K9me2 and H3K9me3, with a higher affinity for the latter residue.^14^ While the role of the H3K9me3 mark is not fully understood, it is thought to be important in cancer development and to regulate apoptosis, DNA repair, splicing, self-renewal and other biological effects.^15^ Experimental inhibitors of KDM4A activity have emerged that suggest a beneficial effect in some cancers.^16^

We sought to define the role of KDM4A for growth in MPM and determine specific dependency for this enzyme. Our data suggest that KDM4A is uniquely elevated in MPM and its functional expression is required for growth and viability in vitro and in vivo, therefore representing a potential vulnerability. Our results further indicate that molecular mechanisms by which KDM4A mediates anti-MPM activity include indirect effects via changing downstream target gene expression and hint at additional targets for drug development.

## Methods

### Immunohistochemistry

TMAs containing tissue cores of 8 normal donors and 53 MPM patients (31 epithelioid, 19 biphasic, 3 sarcomatoid) were reviewed by a certified pathologist. All specimens were obtained through written consent under approval of the Dana-Farber Cancer Institute/Brigham and Women’s Hospital’s Institutional Review Board. MPM patient information (neoadjuvant therapy, histology, sex, age at surgery, smoking history, KDM4A IHC score, staging) is provided in Supplementary Table 1. TMA slides were treated with 3% hydrogen peroxide in methanol (25 °C, 30 min), followed by incubation with 10 mM sodium citrate (pH 6.0) (90 °C, 20 min), and subsequent incubation with 10% normal goat serum in PBS (25 °C, 60 min). After specific staining with anti-KDM4A antibody (A300–861A; Bethyl Laboratories, Montgomery, TX) (4 °C, 20 h), slides were washed with 0.01% Triton X-100 in PBS and specific binding detected with the EnVision system (Agilent, Santa Clara, CA) and the AEC (3-amino-9-ethylcarbazole) substrate (Agilent). Sections were counterstained with hematoxylin (Ricca Chemical, Arlington, TX) and scored according to the intensity of the signal (0, no staining; 1+, weak staining; 2+, moderate staining; 3+, strong staining).

### Cell culture

Cell lines were from ATCC (MSTO (MSTO-211H), H28, H2804 and H2052) and were either used within 6 months of culturing or submitted for cell line authentication within 6 months of use through cell line short tandem repeat (STR) profiling (Molecular Diagnostics Laboratory, Dana-Farber Cancer Institute). Primary-derived cell lines were obtained from primary MPM tissue as described.^17^ Human HEK-293T/17 cells were used for production of lentiviruses and human mesothelial LP9.TERT cells^18^ (kindly provided by Dr. M.R. Ramsey, BWH) were compared to MPM cell lines. In some experiments, cells were treated with KDM4A inhibitors, including PKF118–310 (Sigma–Aldrich), ML324 (Selleckchem) or treated with the cytotoxic chemotherapeutic cisplatin (Santa Cruz Biotech.), the folate anti-metabolite pemetrexed (Selleckchem), the WEE1 inhibitor adavosertib (MK-1775, Selleckchem), the CHK1 inhibitor prexasertib (Selleckchem) or the BH3 mimetics venetoclax (Selleckchem), navitoclax (Selleckchem) and S63845 (Active Biochem Ltd, Hong Kong). Concentrations of viable cells were determined by trypan blue (Sigma–Aldrich, St. Louis, MO) exclusion and cell growth was measured over time with the CellTiter-Glo Luminescent Cell Viability Assay Kit (Promega).

### Immunoblotting

Immunoblotting was performed as described previously using a standard chemiluminescence technique.^19^ Rabbit polyclonal antibodies against KDM4A/JMJD2A (A300–861A, Bethyl Laboratories), KDM4B/JMJD2B (A301–478A, Bethyl Laboratories), KDM4C/JMJD2C (A300–885A, Bethyl Laboratories), KDM4D/JMJD2D (91370, ActifMotif), Histone H3 (A300–823A, Bethyl Laboratories), Histone H3K9me3 (A-4042, EpiGentek), Histone H3K36me3 (A-4036, EpiGentek) or SETD2 (A3194, ABclonal) and mouse monoclonal antibodies against β-actin (12H8; Sigma–Aldrich) were used to measure protein expression.

### RNA interference

Targeted knockdown was performed as described previously,^20^ using lentiviral constructs targeting KDM4A (TRCN0000234910 (#10) and TRCN0000234912 (#12)) and a lentiviral control construct (Sigma–Aldrich).

### Wound healing assay

A wound healing assay was performed on confluent cells in standard 24-well cell culture plates. The monolayer of cells was scratched with a sterile pipette tip and images were obtained immediately and after 24 h of the production of the wound. Wound closure was analyzed using ImageJ (https://ij.imjoy.io/) and expressed as a percentage of wound closure and compared to controls”.

### Xenograft mouse models

In vivo mouse experiments were performed at the Dana-Farber Cancer Institute (Belfer Center for Applied Cancer Science) after approval by the Institutional Animal Care and Use Committee in an AAALAC accredited facility (protocol number 04–111). Female SCID Hairless Outbred (SHO) mice, 6 weeks old, were obtained from Charles River Laboratories (Wilmington, MA) and implanted with 5.0 × 106 MSTO cells with 50% Matrigel (Corning Life Sciences, MA) subcutaneously in the right flank. Tumours were allowed to grow to ~226.5 ± 9.8 mm^3^ (mean ± SEM) in size before randomisation using Studylog software (San Francisco, CA) into a control group with 10 mice and a treatment group with 6 mice. The cages were labeled with cage cards clearly written with the treatment groups after randomisation. The vehicle mice were in separate cages than the compound treated mice. At the time of treatments, only one cage was taken at a time for dosing. The vehicle mice were dosed first. The compound vial was taken out after the dosing of vehicle was completed. Animals were treated with vehicle control (10 μL of 0.5% DMSO in PBS) or PKF118 at 0.70 μg/mouse intratumorally. The animals were dosed for a total of 10 injections (5 injections/weekday). Tumour volumes were determined from caliper measurements by using the formula, Tumour volume = (length × width^2^)/2. Tumour volumes and body weights were measured twice weekly. Animals were euthanised if the tumour volume exceeded 2000 mm^3^ or due to necrotic tumours. Each animal was euthanised by CO^2^ inhalation followed by cervical dislocation. Unpaired *t*-test was performed for each day comparing the vehicle group to the treated group.

### Cell cycle analysis

Cells were fixed with 70% (v/v) ethanol on ice for 30 min and subsequently stained with propidium iodide solution (50 μg/mL propidium iodide, 1% NP40, 10% sodium citrate; Sigma–Aldrich) at 4 °C for at least 15 min. DNA content was quantitated by flow cytometry, and cell cycle distribution was then determined using ModFit LT (Verity Software House, Topsham, ME).

### Apoptosis

The induction of apoptosis was determined in vehicle-treated and control cells using the Annexin-V Fluos staining kit (Roche Diagnostics, Mannheim, Germany) according to the manufacturer’s directions. Annexin-V/PI-positive staining was quantitated by flow cytometry.

### Statistical analysis

For statistical comparison between test and control groups, the Student’s *t*-test was used. Changes were calculated as the percent change relative to the control average. Error bars represent standard deviation (SD) of at least four independent experiments. The cBioPortal.org website^21,22^ was queried for median expression levels of KDM4A in various cancers (TCGA datasets) using identical parameters, as indicated. The Chi-square test for trend was used to determine whether MPM is associated with an increase in immunohistochemistry staining intensity.^23^

### RNA sequencing (RNAseq) analysis

Total RNA was isolated from cells using TRIzol (Ambion by Life Technologies) followed by RNeasy purification (RNeasy kit, Qiagen). Deep sequencing of RNA (RNAseq) and data analyses were performed at the Integrative Genomics Core (City of Hope, Duarte, CA). Briefly, 100 ng of total RNA per sample was subjected to library preparation using the KAPA Stranded mRNA-Seq Kit (KK8421; Kapa Biosystems, Wilmington, MA), followed by sequencing on the Illumina HiSeq 2500 platform. Reads were aligned against the human genome (NCBI build hg19) using TopHat2^24^ and tabulated using HTSeq-count with UCSC known gene annotations (TxDb.Hsapiens.UCSC.hg19.knownGene). *P*-values were calculated from raw counts using edgeR (v.3.20.9), and false discovery rate (FDR) values were calculated using the method of Benjamini and Hochberg.^25^ Gene Set Enrichment Analysis (GSEA)^26,27^ was performed against signature databases (c2.cp.kegg.v6.2.symbols.gmt and h.all.v6.2. symbols.gmt).

## Results

### KDM4A expression is essential for cell growth in models of MPM

**Fig. 1:**
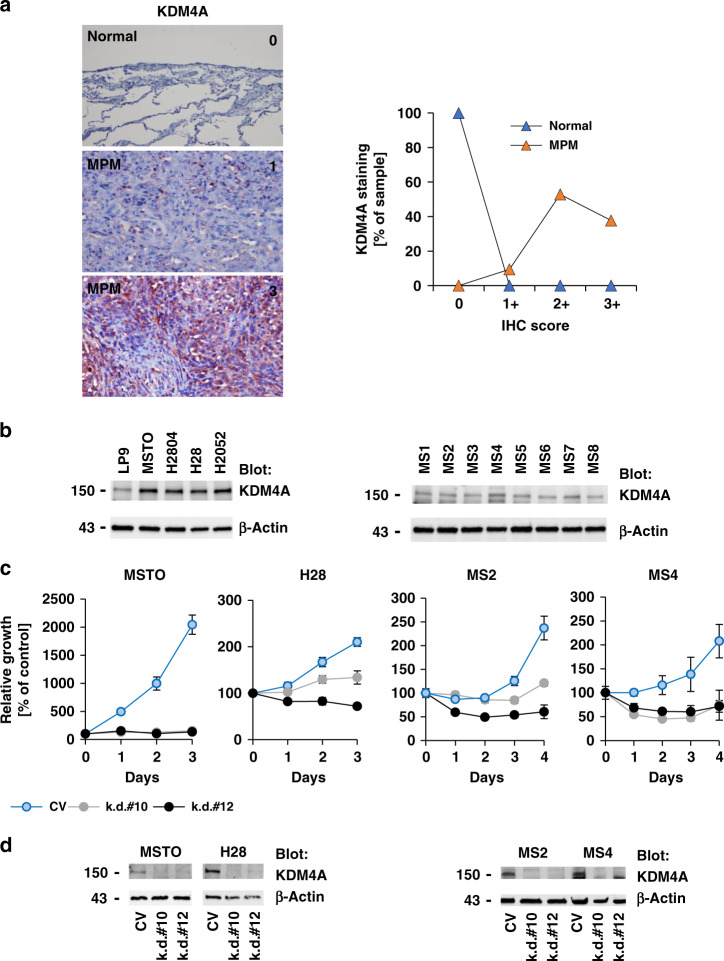
KDM4A expression in MPM is essential for cell growth. **a** Representative IHC images of immunostained TMAs for the expression of KDM4A (left) and analysis of the distribution of IHC scores for normal (*n* = 8) and MPM cores (*n* = 53) (right). **b** Protein expression of KDM4A was determined by immunoblotting in MPM cell lines or MPM specimen-derived cells and compared to expression of β-actin. **c** RNA silencing of KDM4A was performed in cell lines (MSTO, H28) or MPM specimen-derived cells (MS2, MS4) and growth (*n* = 4) was measured, as indicated. **d** Protein expression of KDM4A and β-actin was measured by immunoblotting in cells treated with the indicated RNA interference constructs.

KDM4A levels were evaluated using tissue micro arrays (TMAs), containing a total of 53 MPM (see also Supplementary Table 1) and 8 normal pleural tissue specimens from MPM donors. Tissues were graded based on KDM4A staining intensity. KDM4A protein expression was not detected by IHC in normal mesothelial tissue, but cytoplasmic KDM4A staining was readily found in all MPM tissues (IHC score: 1+: 9.4% (5/53); 2+: 52.8% (28/53); 3+: 37.7% (20/53)) (Fig. 1a). The results suggested highly significant differences in KDM4A levels between normal mesothelial and MPM tissue specimens (*p* = 4.74 × 10^–10^). Similar differences in KDM4A protein levels were also detected in the mesothelial control cell line LP9, with lower levels of KDM4A, compared to the MPM cell lines MSTO, H2804, H28 and H2052 that express higher levels of KDM4A (Fig. 1b, left). Consistent with the above data, KDM4A was also readily detected in MPM cell lines newly derived from surgical MPM tumour specimens (Fig. 1b, right), control primary mesothelial cells are not available for comparison. The protein expression data demonstrated that KDM4A levels are elevated in MPM. We further queried the cBioPortal.org database for a descriptive analysis of the expression of histone lysine demethylase family members and compared their median expression among different types of cancers. Expression levels were ranked and we found KDM4A to have the highest relative expression in MPM, in comparison to other cancer types (Supplementary Fig. S1). Similar to KDM4A, other KDM4 family members were also present in MPM cell lines and may contribute to the regulation of histone methylation. We also found SETD2 expressed in all cell lines tested (Supplementary Fig. S2).

The importance of increased KDM4A expression for cell growth was determined in cell line models of MPM (MSTO/biphasic and H28/epithelioid) using an RNA interference approach with two different hairpins (Fig. 1c, left). The hairpins tested in cell lines significantly suppressed cell growth compared to control infected cells or led only to modest growth after three days in MSTO and H28 (*p* < 0.0005). Similarly, infections of MPM primary-derived cells achieved analogous results compared to those of established cell lines. We observed significant inhibition of cell growth after four days with hairpin #10 or hairpin #12 (*p* < 0.01) compared to cells containing a control vector (Fig. 1c, right). Overall, these models suggest that targeting KDM4A has the potential to block cell growth and reduce viability in MPM. The efficiency of the knockdown was confirmed by immunoblotting (Fig. 1d).

### Inhibition of KDM4A with small molecule drugs reduces growth in models of MPM

**Fig. 2:**
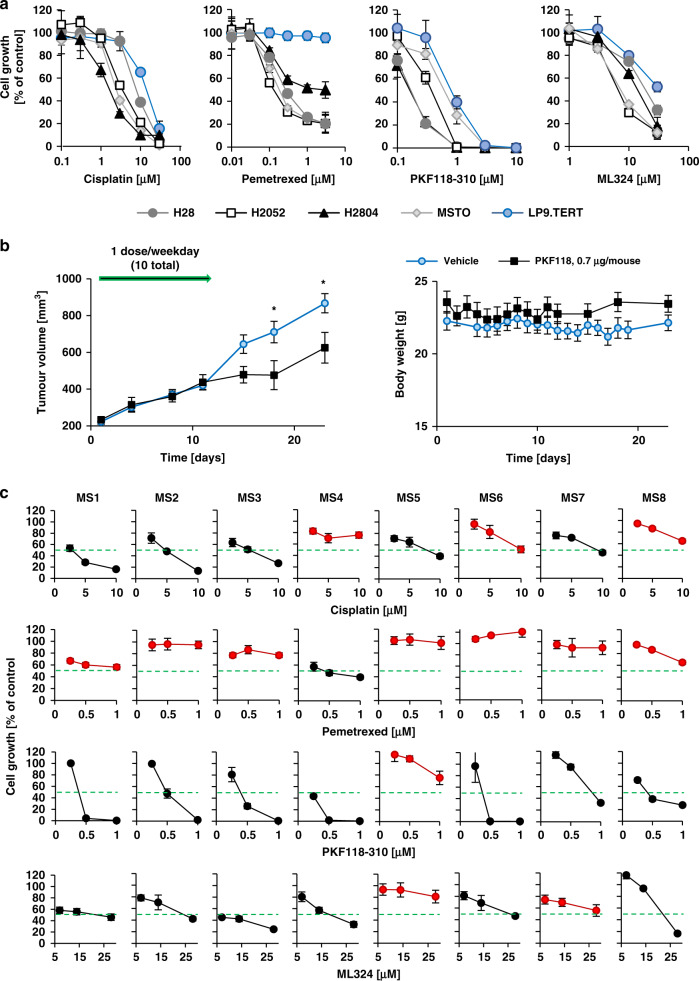
KDM4A inhibitors reduce growth in models of MPM. **a** Cell growth was measured in response to cisplatin, pemetrexed and the KDM4A inhibitors PKF118–310 and ML324 in a 3-day assay (*n* = 4) in the MPM cell lines H28, H2052, H2804 and MSTO, and the non-transformed mesothelial cell line LP9. **b** Mice implanted with MSTO cells were treated with 0.75 μg/mouse PKF118–310 (*n* = 6) or vehicle (*n* = 10). Tumour volume and body weight were monitored (NS not significant). **c** Cell growth was measured in primary-derived MPM cell lines (MS1-MS8) in a 3-day assay (*n* = 4). Cells were treated with the KDM4A inhibitors PKF118–310 and ML324 at the indicated concentrations. Treatments with <50% growth inhibition are indicated in red and the 50% cut-off line is indicated in green.

First-line chemotherapy in patients with MPM involves cisplatin in combination with pemetrexed,^28^ and both drugs are also effective in mesothelioma cell lines H28, H2052, H2804 and MSTO when compared to the mesothelial control cell line LP9 (Fig. 2a). In MPM cell lines, cisplatin (IC_50_ = 1.7–8.5 μM) and pemetrexed (IC_50_ = 0.14–2 μM) lead to a dose-dependent reduction of cell growth with IC_50_ values lower than those for LP9 cells (IC_50_ = 12.1 μM and IC_50_ > 3 μM, respectively). Thus, these results show significantly higher sensitivity of MPM cells versus non-malignant mesothelial cells towards chemotherapeutics (LP9 versus MPM cell lines: 10 μM cisplatin: *p* < 0.001; 1 μM pemetrexed: *p* < 0.001) and constitute a reasonable in vitro model to evaluate drug efficacies. The effective concentrations in MPM cell lines are similar to or below the in vivo plasma concentrations found in patients treated with cisplatin and pemetrexed^29^.

To further complement our RNAi experiments, we tested the small molecule KDM4A inhibitors PKF118–310 and ML324. Both KDM4A inhibitors showed higher sensitivity against MPM cell lines (IC_50_ values: PKF118–310 = 0.2–0.7 μM; ML324 = 7.6–17.9 μM) than against LP9 control cells (IC_50_ values for LP9 cells: PKF118–310 = 0.9 μM; ML324 > 30 μM) (Fig. 2a). For example, significant differences between LP9 and MPM cell lines tested were observed at 1 μM PKF118–310 (*p* < 0.005, except MSTO (*p* = 0.05)) and 30 μM ML324 (*p* < 0.001). The higher sensitivity of MPM cells towards these drugs may suggest a KDM4A pathway dependency that is at least in part distinct from growth and viability signals in LP9 control cells and hint at the potential for a therapeutic window. However, the differences for some models were smaller than for others. In control experiments, we tested whether drug treatment with ML324 and PKF118–310 and targeted knockdown of KDM4A suppressed KDM4A activity in MSTO cells. We found that both approaches increased H3K9me3 and H3K36me3 levels, consistent with reduced activity of KDM4A, as expected (Supplementary Fig. S3)”.

As more highly specific KDM4A inhibitors with favourable in vivo pharmacology have not yet come to fruition,^30^ we tested therefore the efficacy of PKF118–310 as it had been tested previously through intra-tumoral injections in vivo.^31,32^ We used a well-established MSTO xenograft tumour mouse model and injected PKF118–310 within its toxicity limits (Fig. 2b). Reflecting the fact that PKF118–310 was dosed to avoid toxicity (0.7 μg/mouse; 5 injections at weekdays/week), treatment itself did not alter the body weight of the mice (Fig. 2b, right). There was a consistent reduction in tumour mass in the PKF118–310-treated animals from the end of the two-week dosing period that was maintained through day 23 (*p* < 0.03, day 18 and 23, *t*-test) with a final average volume of 867.2 ± 52.0 mm^3^ in control-treated versus 625.1 ± 83.4 mm^3^ in drug-treated mice. Another treatment group at a higher dose (0.85 μg/mouse) that was compared to the same control group and treated at the same time required an intermittent schedule (3 injections/week) with a similar outcome (Supplementary Fig. S4).

We next determined the effect of these compounds on the growth of primary-derived MPM cell lines and compared them to cisplatin- and pemetrexed-treated cells. The experiments were performed at concentrations at the approximate IC_50_ for each drug, and two-fold and four-fold higher (Cisplatin IC_50_ ~2.5 μM; pemetrexed IC_50_ ~0.25 μM; PKF118–310: IC_50_ ~0.25 μM; ML324: IC_50_ ~7 μM) (Fig. 2c). Cells were defined here as insensitive to drug treatment if they did not reach 50% inhibition of cell growth at 4× IC_50_ found in cell lines. Three primary-derived MPM cell lines were found to be insensitive to cisplatin, including MS4, MS6 and MS8. MS4 and MS8 had received cisplatin neoadjuvant treatment, and all but MS4 were found to be insensitive to pemetrexed. On the other hand, we observed variable sensitivity to the KDM4A inhibitors and specimen MS1, MS2, MS3, MS4, MS6 and MS8 were sensitive to inhibition by both PKF118–310 and ML324. The results show that inhibition of KDM4A may be effective on tumour cells that do not respond well to cisplatin, as was observed in specimen MS4 and MS8. To test the hypothesis that cisplatin might show cooperative effects with KDM4A inhibition, we treated primary-derived MPM cell lines with a fixed concentration of cisplatin. In primary-derived MPM cells the cisplatin concentration was held constant and combined with increasing concentrations of ML324 (Supplementary Fig. S5). Combination of cisplatin with ML324 resulted in an increased inhibition of cell growth for the combination relative to each drug alone (*p* < 0.05 at 15 μM ML324), with the exception of specimen MS1, where cisplatin already led to a substantial inhibition of growth.

### KDM4A is required for optimal motility in MPM cells

**Fig. 3:**
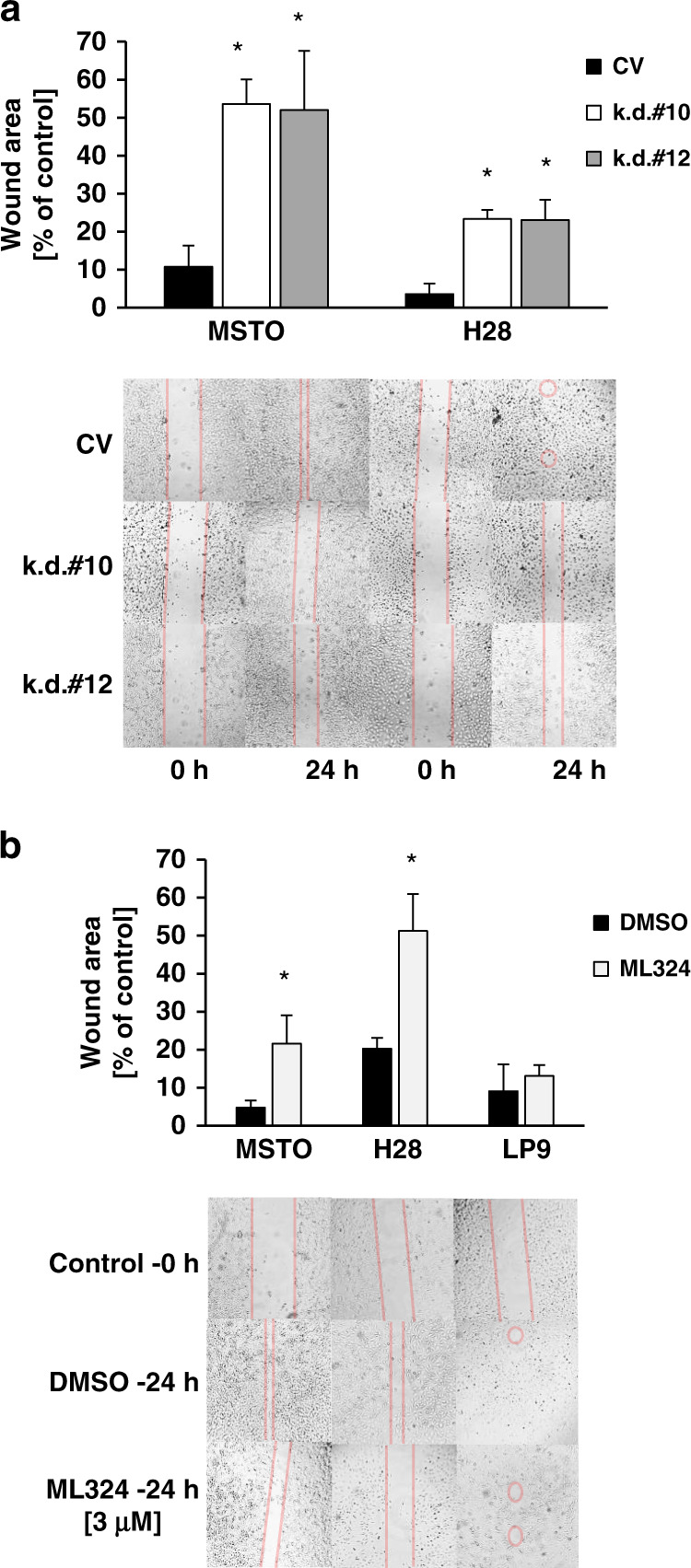
KDM4A knockdown and inhibition with ML324 reduces migration of MSTO and H28 cells in a wound healing assay. Cell cultures were grown to near confluency and wounded after **a** 96 h of KDM4A knockdown or **b** before treatment with ML324 (3 μM) and changes were measured after 24 h, as indicated. Horizontal red lines and marks indicate the areas lacking cells. Data were analyzed with ImageJ and results are expressed as percentage of wound closure and compared to controls (*p < 0.05). Representative images are shown from three (**a**) or four (**b**) independent experiments.

To test whether the anti-tumour effect of KDM4A targeting goes beyond changes in cell growth, we also looked to see whether KDM4A knockdown reduced motility or migration in a standard wound healing assay. Both MSTO and H28 cells showed significantly reduced wound closure with hairpin #10 or hairpin #12 (*p* < 0.05) compared to cells containing a control vector (Fig. 3a). A similar effect could also be achieved with inhibition of KDM4A by ML324 in MSTO and H28 cells. Both MPM cell lines responded to ML324 treatment with significantly reduced wound closure (*p* < 0.05), but not LP9 control cells (Fig. 3b). MPM cell lines were also tested in a 3D spheroid assay in response to ML324 (Supplementary Fig. S6). Whereas MSTO, H2804 and H2052 cells formed spheroid structures that were inhibited in growth (*p* < 0.05) compared to LP9 cells, H28 failed to grow at a reasonable rate in this assay and did not show differences. Thus, targeting KDM4A may affect biological activities, which are associated with transformation that go beyond cell growth.

### Regulation of apoptosis and cell cycle distribution by KDM4A inhibitors

**Fig. 4:**
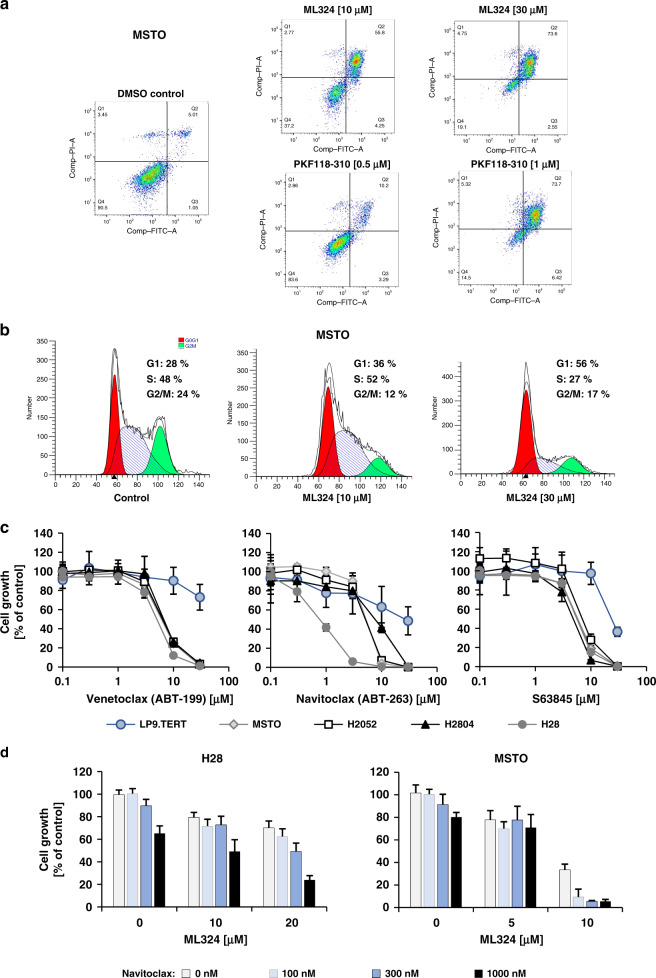
KDM4A inhibitors regulate apoptosis and cell cycle in MPM cell lines. **a** MSTO cells were treated for 24 h with the indicated concentrations of PKF118–310 and ML324 or vehicle, and Annexin-V and propidium iodide staining was determined by flow cytometry (representative experiment shown; *n* = 2). **b** MSTO cells were treated for 24 h with the indicated concentrations of ML324 or vehicle. The percentage of cells in each cell cycle phase was determined by flow cytometry after propidium iodide staining (representative experiment shown; *n* = 2). **c** The indicated MPM cell lines or LP9 cells were treated with the indicated BH3 mimetic, and cell growth was measured after three days (*n* = 4). **d** MPM cell lines were treated with navitoclax in combination with the KDM4A inhibitor ML324 at the indicated concentrations and cell growth was quantitated.

Given the inhibitory effects of PKF118–310 and ML324 on cell growth, we investigated the effects of these drugs on apoptosis and cell cycle distribution in MPM cells. Treatment of MSTO cells with either KDM4A inhibitor, ML324 (10 μM) or PKF118–310 (0.5 μM), led to a prominent increase in apoptosis (Fig. 4a). For direct comparison, the same concentrations were also used for the treatment of MSTO cells with ML324 to evaluate the effect on cell cycle distribution. We observed a dose-dependent increase in G1 phase with 10 μM and 30 μM ML324 (Fig. 4b). The strong induction of apoptosis in PKF118–310 treated cells prevented us from obtaining useful cell cycle distribution data at comparable drug concentrations in MSTO cells at this time point (not shown). The results suggest that G1 cell cycle arrest and induction of apoptosis are early events after drug treatment and that there may be cell-specific differences that lead to changes in the balances between these two events.

Further enhancing pro-apoptotic signals in cancer cells through inhibition of BCL2 family members by BH3 domain mimetic drugs^33^ may also provide an opportunity to enhance KDM4A inhibitor-induced apoptosis. As expected, neither inhibition of BCL2 by venetoclax, inhibition of BCL2, BCL-X_L_ and BCL2-L2 by navitoclax or inhibition of MCL-1 by S63845 had significant effects on cell growth in LP9 cells at concentrations ≤10 μM (Fig. 4c). In addition, >50% inhibition of cell growth in MPM cell lines was accomplished for all drugs at 10 μM. These concentrations are, in general, higher compared to IC_50_ values observed in drug-sensitive cell lines.^34–36^ Only H28 cells showed a higher sensitivity towards navitoclax (IC_50_ = 0.67 μM), suggesting that these cells may be more susceptible to inhibition of BCL-X_L_ or BCL2-L2. Next, we looked to see whether navitoclax cooperates with the inhibition of cell growth by the KDM4A inhibitor ML324. Navitoclax (≤1 μM) was combined in H28 and MSTO cells with ML324 at concentrations close to the respective IC_50_ values of the KDM4A inhibitor in these cell lines (Fig. 4d). The combination of ML324 (≥10 μM in H28 and ≥5 μM in MSTO) with navitoclax exceeded inhibition of either drug alone in both cell lines, suggesting potential cooperativity between these classes of drugs.

### Expression of KDM4A is essential for cell growth and DNA damage repair pathways in models of MPM

**Fig. 5:**
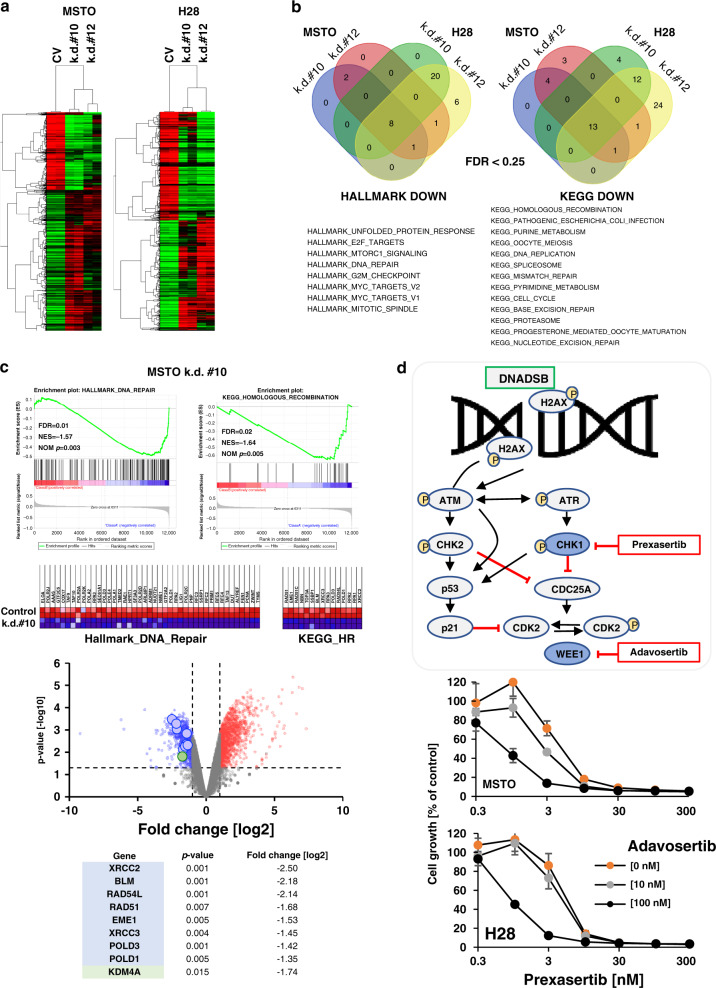
KDM4A is required for cell growth and DNA damage repair pathways. **a** Clustering of genes that were altered in response to KDM4A knockdown (construct #10 and #12), relative to control vector (CV) containing MSTO and H28 cells (*n* = 2). **b** Gene Set Enrichment Analysis (GSEA) showed enrichment with genes in multiple pathways (false discovery rate (FDR) < 0.25). Common Hallmark and KEGG signatures were identified. **c** Depletion of the Hallmark DNA repair signature and the KEGG homologous recombination signature in the GSEA analysis of genes following knockdown of KDM4A construct #10 in MSTO cells were observed. The enriched genes in both signatures are indicated (top panels). Distribution of significantly downregulated genes (blue), upregulated genes (red) (fold change >2 or <0.5; *p* < 0.05) and all other genes (grey) in response to KDM4A knockdown (green circle), are shown relative to their respective *p*-values. The top eight genes within the KEGG homologous repair signature marked on the plot (larger circles) are listed (bottom). **d** Simplified model of the DNA DSB (double-strand break) damage repair signaling pathway (adapted from^38^) (top). Growth was measured in H28 and MSTO cells in a 3-day assay (*n* = 4) in response to the CHK1 inhibitor prexasertib or the WEE1 inhibitor adavosertib (bottom).

As a first step to identify the functional contribution of KDM4A to cell growth in MPM, we performed RNAseq analyses of MSTO and H28 cells in response to KDM4A knockdown using construct #10 and #12. Suppression of KDM4A altered the gene expression profile in both cell lines, and significantly altered genes were identified (*p* < 0.05; >2-fold change in expression) (Fig. 5a and Supplementary Table 2) GSEA (Gene Set Enrichment Analysis) allowed us to identify enriched pathways associated with reduced KDM4A expression in all four models using a Venn diagram (FDR < 0.25). We found depletion of pathways associated with cell growth or viability, but also pathways associated with DNA repair, and others (Fig. 5b). Changes in DNA double-strand break (DSB) repair pathways are of interest here as they may explain some of the genomic abnormalities associated with MPM but they can also independently of classic growth pathways indicate a dysregulation of replication checkpoints.

As an example, GSEA following KDM4A knockdown (construct #10) in MSTO cells resulted in enrichment of Hallmark DNA_Repair (43 enriched genes) and KEGG Homologous_Recombination (15 enriched genes) signatures (Fig. 5c). The top eight genes identified as significantly changed in the volcano plot, are components of the HR (homologous recombination) pathway depicting the role of KDM4A in regulating this pathway. Analysis of archived ChIPseq data showed KDM4A binding at the promoter regions of these eight genes with variable intensity. Whereas the potential for binding to the transcriptional starting site is likely to be similar in different malignancies, the involvement of this enzyme in the regulation of H3K9me3 can differ between malignancies or depend on the genetic background of the sample. For example, KDM4A knockdown in the human AML cell line THP1 shows a distinct increase in the H3K9me3 mark for *XRCC2* and *EME1* at the KDM4A site, compared to control cells. This is consistent with the downregulation of these genes observed in MSTO cells, whereas H3K9me3 did not change for *POLD1* and *POLD3* in this THP1 model (Supplementary Fig. S7).

To see whether specific proteins in the HR DNA double-strand break (DSB) repair pathway define KDM4A-dependent growth and thereby create a therapeutic vulnerability, we explored targeting molecules within the DNA DSB repair pathway, downstream of ATM and ATR kinases (see for review^37^) (Fig. 5d, top). DSBs allow ATM to activate ATR-CHK1 to initiate DNA repair and regulate replication checkpoints. The checkpoint kinase CHK1 can be inhibited by prexasertib and controls cell cycle progression from G1 to S phase through degradation of the CDC25A phosphatase.^38^ WEE1 is a regulator of CDK2 function, downstream of the CHK1-CDC25A axis and can be inhibited by adavosertib.^38^ Indeed, our data demonstrate that adavosertib is more effective in MSTO and H28 cells compared to LP9 control cells (Supplementary Fig. S8). The data also demonstrate that adavosertib can cooperate with prexasertib in MPM cell lines (Fig. 5d, bottom). For example, 10 nM prexasertib by itself was sufficient to suppress growth by >80% (*p* < 0.0005) and a less effective concentration of 3 nM prexasertib led to a similar outcome in the presence of 100 nM MK-1775 (*p* < 0.0005). The results not only support an important role for KDM4A and dependent pathways in growth but also inversely match previously described negative functions of SETD2.^39^

## Discussion

Our analysis suggests that high expression of KDM4A contributes to dysregulation within the H3 lysine methylation pathway. Even though the differences in KDM4A expression are significant, it would now be interesting to evaluate a larger cohort of MPM patients with matching normal tissue to get a better sense of the extent of these differences. Our immunohistochemistry data in conjunction with the RNA interference experiments demonstrate that high expression of KDM4A is required for optimal MPM cell growth. Previous data suggested that KDM4A inhibitors regulate growth through inhibition of cell cycle progression and induction of apoptosis.^40,41^ Our data with the KDM4A inhibitors ML324 and PKF118–310 are consistent with these results. Highly specific small molecule drugs that exclusively target KDM4A and are suitable for clinical trials are not yet available and one of our primary future goals will be to identify and optimise small molecule compounds that can effectively and specifically target KDM4A in vivo. Nevertheless, our RNA interference experiments in MPM cell lines and MPM specimen-derived cells go beyond these findings. We not only demonstrate sensitivity towards KDM4A inhibition but also show a clear pathway dependency for the maintenance of cell growth. Thus, alteration within this pathway may extend beyond normal function and trigger aberrant dependency of MPM cells towards KDM4A.^14,16^

The reliance of MPM on the SETD2/KDM4A pathway does not appear to be universal and KDM4A expression alone may not define responsiveness to inhibitors, as we already have shown differences in sensitivities among cell lines as well as MPM specimen-derived cells. A primary function of KDM4 family members is to control the levels of H3K9 methylation,^14,16^ and it would be interesting to know whether this modification has a role in self-renewal of cancer stem cells or growth of bulk cancer cells. We have initiated a more straightforward approach by looking for genes that are altered by KDM4A knockdown through RNA sequencing. Our results suggest KDM4A is involved in pathways traditionally associated with cell growth (G2M_Checkpoint, Mitotic_Spindle, DNA_Replication, Cell_Cycle), DNA damage repair (DNA_Repair Homologous_Recombination, Mismatch_Repair, Base_Excision_Repair, Nucleotide_Excision_Repair), RNA splicing (Spliceosome) and others. These results are consistent with the negative role of SETD2 on these pathway^39^ and like SETD2, KDM4A has been suggested to be involved in DNA repair, transcription initiation, recombination and tumour growth in some models.^14^ Based on this, in the future it should be possible to generate KDM4A expression signature profiles and identify patients that are particularly dependent on these pathways.

The regulation of cell growth by KDM4A may be linked to mechanisms that are traditionally not associated with proliferation. Loss of SETD2 is known to create vulnerabilities in some cancers as it decreased levels of the RRM2 ribonucleotide reductase subunit, which can be further reduced by the WEE1 inhibitor, adavosertib, leading to a depletion of the dNTP pool and S phase arrest.^42^ Similarly, our data showed a 3–8-fold reduction in RRM2 levels with KDM4A knockdown. Thus, functional regulation of the WEE1 pathway and related dependencies may be linked in part to the dysregulation of DNA DSB repair mechanisms. This is of interest since CHK1 inhibition has been suggested to synergise with WEE1 inhibition by forcing cells into abnormal mitosis and subsequent cell death.^43^ Consistent with this model, our data in MSTO and H28 cells demonstrate that WEE1 inhibition with adavosertib can cooperate with CHK1 inhibition by prexasertib in MPM cell lines. Additional in-depth studies are needed to evaluate the impact of KDM4A-dependent DNA repair on the fidelity of these processes in particular as it relates to the balance between high fidelity homologous recombination and error-prone non-homologous end joining.

As drug resistance is a significant problem in MPM, our combination experiments are of particular interest. Our results suggest that KDM4A-targeting small molecule drugs can be efficacious independent of responsiveness to cisplatin. Another way of enhancing KDM4A inhibition would be to sensitise cells to the KDM4A-mediated pro-apoptotic response. We tested the specific BCL2 family inhibitor venetoclax, as well as navitoclax and S63845. Only navitoclax showed single agent activity in H28 cells, suggesting a dependency of these cells for either BCL-X_L_ or BCL2-L2 but not BCL2 or MCL-1.^36,44^ Interestingly, our data showed that inhibition by navitoclax also cooperated with KDM4A inhibition in MSTO cells, even though navitoclax had little single agent activity. A closer look at navitoclax and the pathway affected by its activity in MPM may be warranted. Additional regression analysis for BCL2-L2 showed that there was a positive correlation between expression and survival post-surgery (*p* < 0.001) in a previously published dataset.^10^ No significant correlation was found between survival and expression of BCL2 or BCL-X_L_ (BCL2-L1) (not shown). Next, it would be interesting to determine the exact involvement of either BCL-X_L_ or BCL2-L2 in MPM. Combination of navitoclax with pro-apoptotic agents in MPM may provide a new venue for targeted approaches and allow for more efficient combination therapy. Overall, KDM4A exhibits features in our models that are normally associated with transforming oncogenes, making it a promising target for drug development and further study.

## Supplementary information

Supplementary Figures and Table S1

Supplementary Table S2

ARRIVE Checklist

## Data Availability

Additional data from this study can be found in the supplementary section.
