# Supplementary material for: Essential role of the histone lysine demethylase KDM4A in the biology of malignant pleural mesothelioma (MPM)
Source: Br J Cancer. 2021 Jun 4;125(4):582–92. doi: 10.1038/s41416-021-01441-7 (PMC8368004; doi:10.1038/s41416-021-01441-7)
Supplement: Supplementary file 1 — Supplementary Figures and Table S1 [file 41416_2021_1441_MOESM1_ESM.pdf]

Supplementary Figures

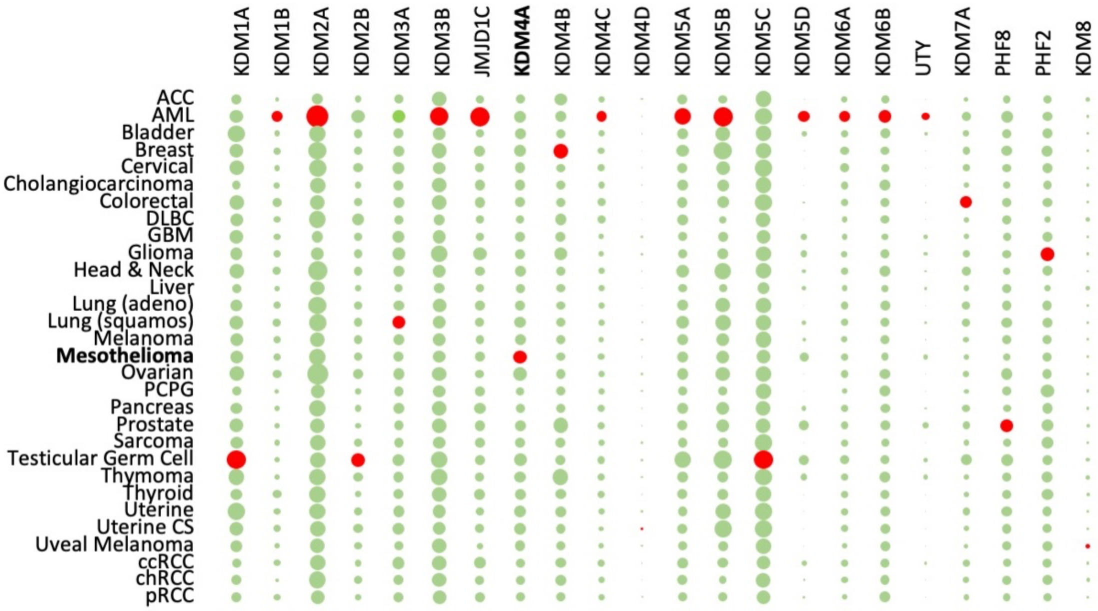

Figure S1. Expression of lysine demethylases in various cancers. A, Median expression of lysine demethylase family members (excluding KDM4E/F) was compared amongst various cancers, as indicated. The area of the circle represents the relative expression with the highest value per family member marked in red.

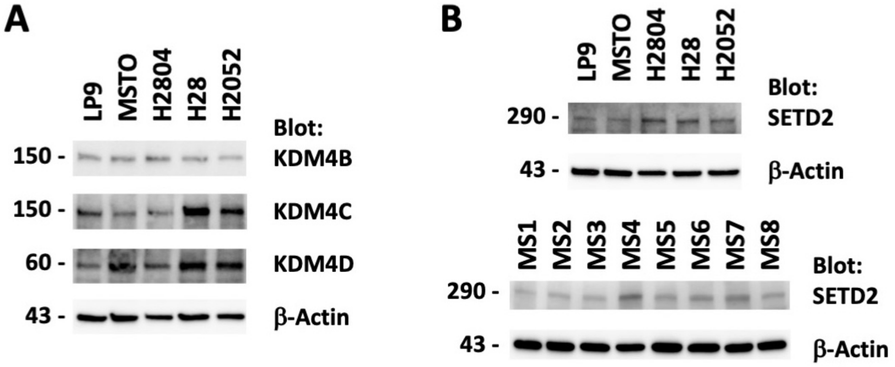

Figure S2. Expression of histone H3 lysine modifying enzymes in MPM cell lines. A, Protein expression of the KDM4 family members 4B, 4C, and 4D was determined in cell lines, as indicated. **B**, Protein expression of SETD2 was determined in LP9 cells, MPM cell lines and primary-derived cell lines. Protein expression of β-actin was used as a loading control.

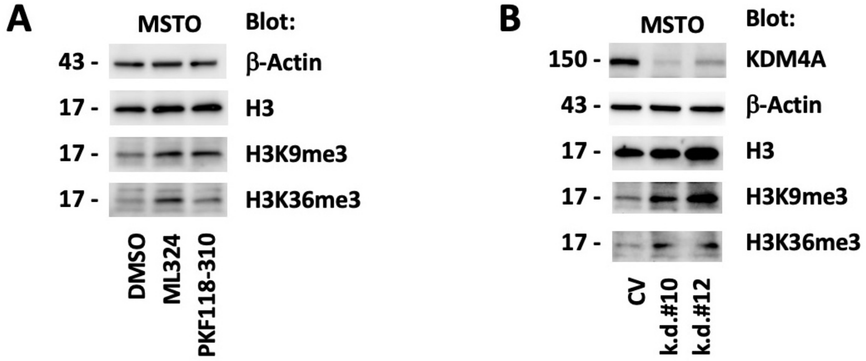

Figure S3. Histone H3 lysine modification in MPM cell lines. Protein expression of the histone H3, H3K9me3 and H3K36me3 was determined in MPM cell lines in RIPA buffer cell extracts, in response to (**A**) drug treatment with ML324 (20 µM) and PKF118-310 (0.5 µM) for 18h or (**B**) KDM4A knockdown with construct #10 and #12, as indicated.

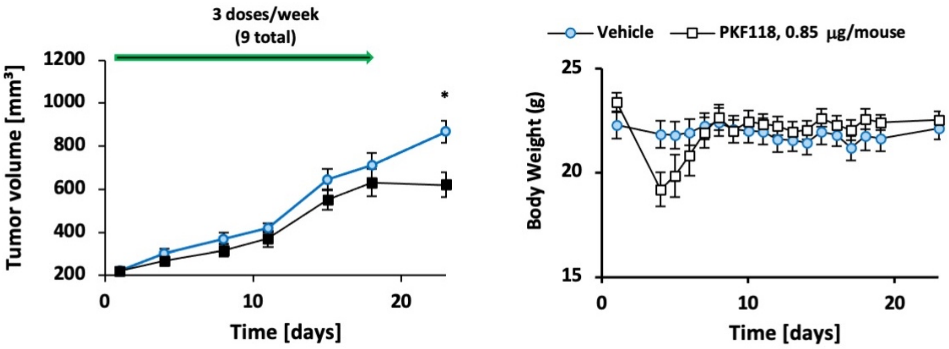

Figure S4. Mice implanted with MSTO cells were treated with 0.8 µg/mouse PKF118-310 (n=6) or vehicle (n=10), as indicated. Tumor volume and body weight were monitored (NS=not significant).

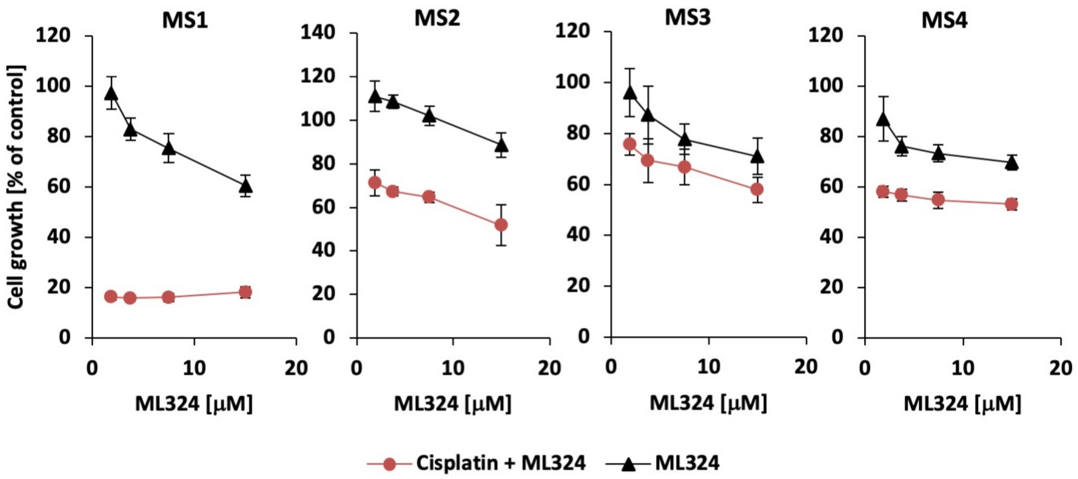

Figure S5. Cell growth of primary-derived MPM cell lines in response to cisplatin and ML324. Cell growth was measured in a three-day assay (n=4) in primary-derived MPM cell lines (MS1-MS4) in response to cisplatin and the KDM4A inhibitor ML324, as indicated.

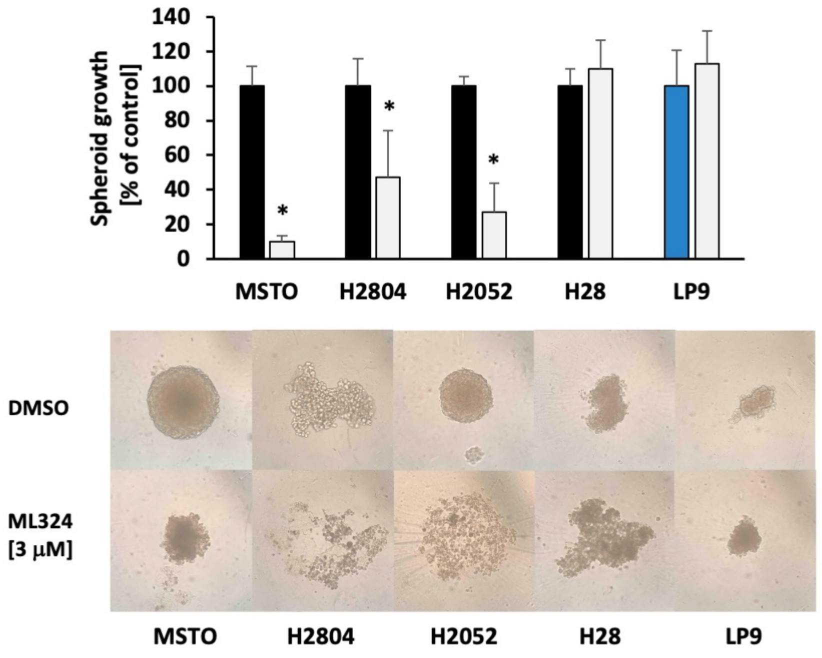

Figure S6. 3D spheroid culture of MPM cell lines. Growth of MPM cell lines and LP9 control cells was determined in low adherence cell culture plates (Corning, Cat No. 4515) and measured with the CellTiter-Glo Luminescent Cell Viability Assay Kit (Promega), top panel. *Significant differences are indicated ($p<0.05$; n=4). Representative images are shown, bottom panel.

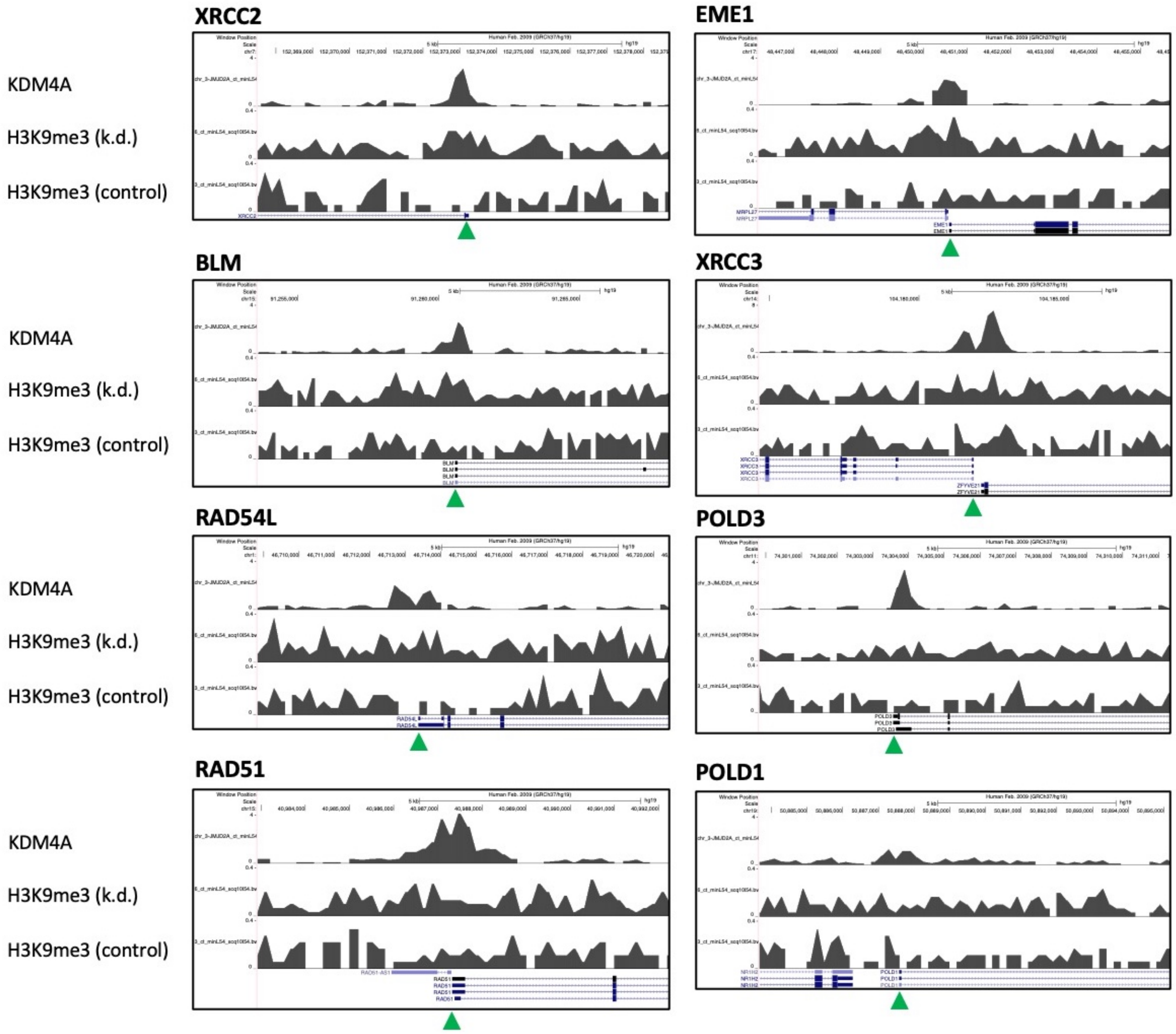

Figure S7. Snapshot of KDM4A occupancy and histone H3K9me3 marks. KDM4A ChIPseq data from wild-type THP1 cells (GSM3572797) and H3K9me3 ChIPseq data from THP1 with non-target control (GSM3572793) or KDM4A knock-down (k.d.) (GSM3572796) were analyzed for KDM4A occupancy or enrichment of H3K9me3 signals around the transcriptional starting site (green triangle) at genes involved in DNA repair using the UCSC Genome Browser (https://genome.ucsc.edu/cgi-bin/hgGateway), as indicated.

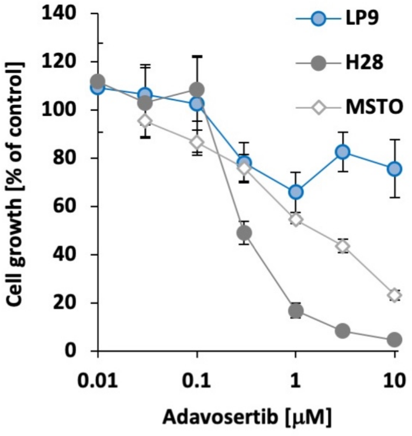

Figure S8. Cell growth of LP9, H28 and MSTO cells in response to adavosertib. Cell growth was measured in a three-day assay (n=4) in response to the WEE1 inhibitor adavosertib in MPM cell lines and compared to the mesothelial control cell line LP9, as indicated.

Neoadjuvant therapy	Histology	Sex	Surgery	Age (years)	Smoking	KDM4A IHC	Staging
-	Epithelioid	Male	DBK	61	Yes	1	*
-	Sarcomatoid	Male	EPP	56	Yes	2	T3N0
-	Epithelioid	Male	EPP	63	Yes	2	T4N2
+	Biphasic	Female	EPP	72	Yes	2	T4N2
+	Biphasic	Female	EPP	71	No	2	T2N2
-	Epithelioid	Male	EPP	63	No	2	T0N2
-	Epithelioid	Female	EPP	68	Yes	2	T4N2
+	Epithelioid	Male	EPP	55	Yes	2	T4N0
-	Epithelioid	Male	PDC	78	No	2	*
-	Biphasic	Male	PDC	77	Yes	2	*
-	Biphasic	Male	PDC	76	Yes	2	*
-	Biphasic	Male	PDC	82	Yes	1	*
-	Epithelioid	Male	PDC	75	Yes	2	*
-	Epithelioid	Male	PDC	58	No	3	*
-	Biphasic	Male	PDC	77	Yes	3	*
-	Epithelioid	Male	EPP	55	Yes	1	T2NO
-	Biphasic	Male	PDC	83	No	3	*
-	Epithelioid	Male	PDC	72	No	2	*
-	Epithelioid	Male	PDC	77	No	2	*
-	Epithelioid	Male	DBK	73	Yes	3	T3NO
-	Biphasic	Male	EPP	75	Yes	2	*
-	Epithelioid	Male	PDC	73	Yes	1	*
-	Biphasic	Male	PDC	53	Yes	2	*
-	Epithelioid	Male	PDC	53	Yes	2	*
-	Biphasic	Male	EPP	49	Yes	2	T3N2
+	Epithelioid	Male	EPP	52	No	2	T4N2
-	Biphasic	Male	EPP	70	Yes	3	T3N0
-	Epithelioid	Male	PDC	59	Yes	2	*
-	Epithelioid	Female	EPP	59	Yes	1	T2N1
-	Biphasic	Male	EPP	61	Yes	2	T4N2
-	Epithelioid	Male	EPP	39	Yes	2	T2N0
-	Epithelioid	Male	EPP	55	No	3	T4N1
-	Epithelioid	Female	EPP	55	Yes	3	T2NO
+	Epithelioid	Female	EPP	39	No	3	T2N2
-	Biphasic	Male	EPP	55	No	2	T3N0
+	Biphasic	Male	EPP	62	Yes	2	T3NO
-	Epithelioid	Male	PDC	63	Yes	2	*
+	Biphasic	Male	EPP	64	No	2	T4N2
+	Biphasic	Male	EPP	62	Yes	2	T4N2
-	Epithelioid	Male	EPP	38	No	3	T3N2
-	Biphasic	Male	PDC	64	Yes	2	*
+	Biphasic	Male	EPP	64	No	3	T3N2
+	Epithelioid	Female	EPP	66	No	2	T4NO
-	Epithelioid	Female	EPP	63	Yes	3	T4N2
-	Epithelioid	Male	PDC	84	No	3	*
-	Sarcomatoid	Male	EPP	70	No	2	T4N0
+	Epithelioid	Male	PDC	64	Yes	3	*
-	Biphasic	Male	EPP	59	Yes	3	T4N2
-	Epithelioid	Male	PDC	69	No	3	*
-	Sarcomatoid	Male	PDC	67	Yes	3	*
-	Epithelioid	Male	EPP	34	Yes	3	T3NO
-	Biphasic	Male	EPP	63	Yes	3	T4NO
-	Epithelioid	Female	EPP	54	No	3	T4N1

Total MPM	53
KDM4A IHC "1"	5
KDM4A IHC "2"	29
KDM4A IHC "3"	19
Epithelioid	30
Biphasic	20
Sarcomatoid	3
Neoadjuvant therapy	11
Median age	63

* partial resection that did not allow for accurate staging

Table S1. Patient information.
